# Performance of Global-Appearance Descriptors in Map Building and Localization Using Omnidirectional Vision

**DOI:** 10.3390/s140203033

**Published:** 2014-02-14

**Authors:** Luis Payá, Francisco Amorós, Lorenzo Fernández, Oscar Reinoso

**Affiliations:** Departamento de Ingeniería de Sistemas y Automática, Miguel Hernández University, Avda. de la Universidad s/n, Elche (Alicante), Spain; E-Mails: famoros@umh.es (F.A.); l.fernandez@umh.es (L.F); o.reinoso@umh.es (O.R.)

**Keywords:** omnidirectional vision sensor, global appearance descriptors, map building, localization, image recovering, particle filter

## Abstract

Map building and localization are two crucial abilities that autonomous robots must develop. Vision sensors have become a widespread option to solve these problems. When using this kind of sensors, the robot must extract the necessary information from the scenes to build a representation of the environment where it has to move and to estimate its position and orientation with robustness. The techniques based on the global appearance of the scenes constitute one of the possible approaches to extract this information. They consist in representing each scene using only one descriptor which gathers global information from the scene. These techniques present some advantages comparing to other classical descriptors, based on the extraction of local features. However, it is important a good configuration of the parameters to reach a compromise between computational cost and accuracy. In this paper we make an exhaustive comparison among some global appearance descriptors to solve the mapping and localization problem. With this aim, we make use of several image sets captured in indoor environments under realistic working conditions. The datasets have been collected using an omnidirectional vision sensor mounted on the robot.

## 1. Introduction

During the last years, omnidirectional cameras have become a widespread sensor in mobile robotics mapping and localization tasks, thanks to their relative low cost and the richness of the information they provide us with. When we mount one of these cameras on a robot, this information can be used to build a model or map of the environment and to estimate the position and the orientation of the robot within this map. There are many approaches to carry out these tasks. Amongst them, global-appearance techniques represent a very promising alternative. These techniques lead to conceptually simple algorithms since each image is represented by only one descriptor and the mapping and localization processes can be carried out by comparing these global descriptors. They also present some advantages over classical local features extraction and description methods, especially in dynamic and non structured environments, where it is difficult to extract and describe stable landmarks. However, when we apply them to solve a real time mapping and localization problem, some restrictions must be taken into account during the design of the algorithms.

In this work, a review and comparison is made taking into consideration different methods to extract the most relevant information from a set of images, based on their global-appearance. We propose to use several descriptors, based on Discrete Fourier Transform, Principal Components Analysis, Histograms of Oriented Gradients, and *gist* of scenes. We adapt and optimize these descriptors to be used with omnidirectional information, and we study how several parameters affect their performance, their invariance against rotations of the robot on the ground plane, their computational requirements and the accuracy in localization they offer. Some of these descriptors have not been previously used in the field of robotics mapping and localization.

For this purpose, we present the results of a set of experiments developed with several large databases composed of panoramic images, captured in different real indoor environments. We also study the effect of common situations that usually happen in real applications:

- Changes in lighting conditions, due to the fact that the robot navigates within the environment at different times of day and with presence or not of artificial illumination.
- Occlusions. People moving around the robot can temporary appear in the images, occluding part of them.
- Noise produced by the vision sensor.
- Visual aliasing. In indoors environments, it usually happens that two images captured from two distant points have a similar appearance.

The main objective is to demonstrate the applicability of the different descriptors to robotic mapping and localization tasks, and to measure their goodness and computational requirements. The experimental setup allows us to validate them and to make a detailed comparative analysis of the different techniques. We prove that it is possible to create an optimal model of the environment where the robot can estimate its position and orientation in real time and with accuracy, using just the information provided by an omnidirectional vision sensor.

## 2. Topological Mapping and Localization through Global Visual Appearance

Over last years, omnidirectional vision sensors have gained popularity thanks to the big quantity of information they provide, as they have a 360 degrees field of view around the robot; the stability of the features that appear in the images, since they last longer in the field of view as the robot moves; their relatively low cost comparing with other sensors and their low power consumption. These sensors are usually composed of a conventional camera and a convex spherical, parabolic or hyperbolic mirror (catadioptric system). The visual information can be represented using different projections: omnidirectional, panoramic of bird-eye view [1]. In this work, we make use of the panoramic representation since it contains enough information to estimate the position and the orientation of the robot when its movements are restricted to the ground plane. Many authors have studied the use of this kind of images both in mapping and localization tasks. The high quantity of information they contain make it necessary to use some process to extract the most relevant and useful information from the scenes to solve these problems. The solutions to extract such information can be categorized in two approaches: local feature extraction and global appearance solutions.

The first approach consists in extracting a limited number of relevant local features (such as points, lines or regions) and describing them using an invariant descriptor. Amongst the feature extraction and description methods we can highlight *SIFT* (Scale Invariant Feature Transform) [2] and *SURF* (Speeded Up Robust Features) [3], which provide us with invariant features against changes in scale, orientation, lighting conditions and camera point of view. Both methods have become popular in map creation and localization of mobile robots. For example, Angeli *et al.* [4] make use of *SIFT* features to solve the SLAM and global localization problems, and Valgren *et al.* [5] and Murillo *et al.* [6] make use of *SURF* features extracted from omnidirectional images to find the position of a robot in a previously created map.

The second approach works with each scene as a whole, without extracting any local information. Each image is represented by an only descriptor. These approaches have advantages in dynamic and unstructured environments where it is difficult to extract stable landmarks from the scenes. The main disadvantage is the high memory and time requirements to store the visual information and to compare the descriptors. The current methods for image description and compression allow us to optimize the size of the databases and to carry out the localization process with a relative computational efficiency.

The use of global appearance descriptors is an alternative to the classical methods based on the extraction and description of local features or landmarks. These approaches lead to conceptually simpler algorithms thus they constitute a systematic and intuitive alternative to solve the map building and localization problems. Usually, these approaches are used to build topological maps, which do not include any metric information. In these maps, the environment is often represented by a graph where nodes are images that symbolize distinctive places and links are the connectivity relationships between that places [4].

The key point of the global appearance approach is the description algorithm. Several alternatives can be found in the literature on this topic. Some authors make use of the Principal Components Analysis (PCA) to create visual models with mobile robots ([7,8]). This approach considers images as multidimensional data that can be projected in a new space with a lower dimensionality, retaining most of the information. Other authors make use of the Discrete Fourier Transform (DFT) to extract the most relevant information from the scenes. When working with panoramic images, we can use both the 2D DFT [9] or the Fourier Signature (FS), defined in [10]. The resulting descriptor is able to concentrate most of the information in a lower number of components. Comparing to the classical PCA approaches, the DFT descriptors are invariant against rotations on the ground plane, their computational cost is relatively low and each scene descriptor can be computed independently on the rest of images. Finally, other authors have described the scenes based on the gradient magnitude or orientation. As an example, Kosecka *et al.* [11] make use of a gradient histogram to create a topological map and localize the robot.

We have not found in the related literature any work which makes a deep comparison between global description techniques. In this work we have selected several of the most relevant techniques. We have adapted some of them to describe panoramic scenes. We have also tested their performance depending on their main parameters and we have made a comparative evaluation among them. This comparison has been carried out from several points of view: we have tested them as a tool to solve the mapping and the localization problems (both global localization and probabilistic localization) and we have also taken into account the most relevant phenomena than usually happen in a real application: camera occlusions, noise, changes in lighting conditions and visual aliasing. All the tests have been carried out with two large sets of images captured under real working conditions.

The rest of the paper is organized as follows: in the next section we make a review of the main techniques to globally describe scenes. Section 4 formalizes the implementation of the description techniques to optimally solve mapping and localization tasks when we use panoramic scenes. Then, Section 5 presents the experimental setup, the images databases we have used and the results of the experiments. The work finishes with the discussion and the conclusion sections.

## 3. Global Appearance Descriptors. State of the Art

In this section we firstly make a general description of the map building and localization processes using the global appearance of scenes and secondly we revise the most relevant techniques for image description.

To solve the map building and localization problem using the global appearance of visual information, the first step consists in deciding how to represent such information. Working directly with the pixels of the images would be computationally very expensive. This way, first we will study some ways to globally describe the information in the scenes. To study the viability of these descriptors in map building and localization, we decompose the experimentation in two steps (1) learning and (2) validation.

- *Learning*. A model (or map) of the environment is created. The robot captures a set of images, describes each one with a descriptor and establishes some relationships among the images using the information in the descriptors to build the map.
- *Validation*. The robot captures an image from an unknown position, builds its descriptor and compares it with the descriptors stored in the previously learned model. As a result, the position and orientation of the robot can be estimated.

In the first step, the robot is guided in a teleoperated way, through the environment to map. During this step, the robot acquires a set of omnidirectional images. We then compute the panoramic scenes and as a result we get the set *I* = {*i*_1_, *i*_2_, …, *i_n_*} where *i_j_* ∈ ℝ^*N_x_*^^×^^*N_y_*^ represents each panoramic image.

From this set of images, a set of global descriptors is computed, one per original scene. As a result, the model of the environment is composed of the set of descriptors *D* = {*d*_1_, *d*_2_, …, *d_n_*} where, in general, *d_j_* ∈ ℂ^*M_x_*^^×^^*M_y_*^. Each one of these descriptors represents the main information in each scene. They should present some properties to be efficient in map creation and localization tasks:

- Each descriptor should contain the main information in the original scene with a lower dimension *M_x_* × *M_y_* ≪ *N_x_* × *N_y_* (compression effect).
- There should exist a correspondence between distance among descriptors and geometric distance between the points where the images were captured, *i.e.*, two images that have been captured frome close points should have similar descriptors, and as geometric distance increases, descriptors distances should do too.
- The descriptors should present robustness against some usual situations in mobile robots applications: occlusions in the scenes, changes in the lighting conditions, noise, *etc*.
- The computational cost to compute the descriptor should be lower enough to allow the robot localizing itself in real time.
- It is recommendable that the descriptors can be built incrementally, *i.e.*, each scene should be described independently of the rest of images. This permits building the map online, as the robot is exploring the environment.
- It is necessary that the descriptor includes some information about the orientation the robot had when capturing the image. This means that if a robot captures two images from near points in different orientations, the descriptors should allow us to compute the relative orientation.

In the next subsections we present the main description methods existing in the literature on this topic and their main properties.

### 3.1. Discrete Fourier Transform

The Discrete Fourier Transform of an image can be defined as:

(1) $$I(u,\upsilon )=\sum_{x=0}^{N_{x}-1}\sum_{y=0}^{N_{y}-1}i(x,y)\cdot h(x,y)\cdot e^{-j2\pi (ux+\upsilon y)}=A(u,\upsilon )\cdot e^{j\Phi (u,\upsilon )}$$

where *i*(*x*, *y*) is the intensity distribution of the scene with respect to the spatial variables (*x*, *y*) and (*u*, *υ*) are the frequency variables (cycles/pixel). *h*(*x*, *y*) is a window to reduce the effects of the discontinuity at the beginning and at the end of the image. The complex resulting function *I*(*u*, *υ*) can be decomposed in two real components, the amplitude spectrum *A*(*u*, *υ*) = |*I*(*u*, *υ*)|, which contains non localized information about the structure of the scene (orientation, smoothness, length… of the contours) and the argument Φ(*u*, *υ*), which has information about the local properties of the scene (shape and position of the components of the scene) [12].

Taking these facts into account, the amplitude spectrum can be used as a global descriptor of the scene, as it contains information about the dominant structural patterns and it is invariant with respect to the position of the objects. Some authors have shown how this kind of non-localized information is relevant to solve simple classification tasks [13].

However, this kind of descriptors which are purely based on the 2D-DFT do not contain any information about the spatial relationships between the main structures in the image. To have a complete description of the appearance of the scene it is necessary to include such information. A possible option based on the 2D-DFT is presented in [14]. They make use of a Windowed Fourier Transform over a set of localizations uniformly distributed on the scene. When working with panoramic scenes, a second option, named *Fourier Signature (FS)*, is suggested in [10]. It consists in computing the one-dimensional DFT of each row in the original panoramic scene. Compared to the rest of the Fourier-based methods, the advantages of FS are its simplicity, low computational cost and the fact that it exploits well the invariance against ground-plane rotations using panoramic images.

Taking a panoramic image *i_j_*(*x*, *y*) ∈ ℝ^*N_x_*^^×^^*N_y_*^ as our starting-point, after computing the FS we arrive to a new matrix *d_j_*(*u*, *y*) ∈ ℂ^*N_x_*^^×^^*N_y_*^ where the most important information is concentrated in the low frequency components from each row. This way, we can retain just the *k*_1_ first columns in the signature (*k*_1_ < *N_y_*) (compression effect). The matrix *d_j_*(*u*, *y*) can also be separated in a magnitude matrix *A_j_*(*u*, *y*) = |*d_j_*(*u*, *y*) | and an argument matrix Φ*_j_*(*u*, *y*).

The matrix *A_j_*(*u*, *y*) presents rotational invariance when working with panoramic images. Thanks to the shift theorem (Equation (2)), it is possible to prove that if each row of the original image is represented by the sequence {*a_n_*} and each row of the rotated image by {*a*_*n*_–_*q*_} (being *q* the amount of shift), when the Discrete Fourier Transform of the shifted sequence is computed, we obtain the same amplitudes *A_k_* than in the non-shifted sequence, and there is only a phase change, proportional to the amount of shift *q*.

(2) $$\begin{array}{ll} F[\{a_{n-q}\}]=A_{k}\cdot e^{\left(-j\frac{2\pi ql}{N_{y}}\right)}; & l=0,\ldots ,N_{y}-1 \end{array}$$

Thanks to this property, the estimation of the robot position and the orientation can be made separately. Basically, we first we compute the Fourier Signature and we retain the first *k*_1_ columns, *d_j_*(*u*, *y*) ∈ ℂ^*N_x_*^^×^^*k*1^, then we compute the magnitude matrix and we use it to estimate the position of the robot and then we compute the argument matrix and we use it to estimate the orientation of the robot. Also, this is an inherently incremental method as the descriptor of each image can be computed independently on the rest of images.

### 3.2. Principal Components Analysis

When we have a set of panoramic images *i_j_*(*x*, *y*) ∈ ℝ^*N_x_*^^×^^*N_y_*^, *j* = 1, …, *n*, each image can be considered as a data vector that falls in a space with *N_x_* · *N_y_* dimensions *x⃗_j_*(*i*) ∈ ℝ^*N_x_*^^×^^*N_y_*^, *j* = 1, …, *n*, However these vectors have been generated from a process with just three degrees of freedom (position and orientation of the robot on the ground plane). As these data are highly correlated, the philosophy of Principal Components Analysis (PCA) consists in carrying out a dimensionality reduction with the goal of retaining the most relevant information from each scene.

Using the classical formulation of PCA, as exposed in [15] and [16], we can project the set of data vectors (images) *x⃗_j_*(*i*) ∈ ℝ^*N_x_*^^×^^*N_y_*^^×1^, *j* = 1, …, *n*, being *n* the number of images and *N_x_* · *N_y_* the number of pixels in each image, *n* ≪ *N_x_* · *N_y_*, into a set of feature vectors named *projections of the images p⃗_j_*(*i*) ∈*^K^*^×1^ *j* = 1, …, *n*, being *K* the PCA features that contain the most relevant information from the image *K* ≤ *n*. This projection is computed with the expression: **P** = **V***^T^* · **X**, where **X** is the data matrix, composed of the data vectors arranged in columns and **P** is the projection matrix, containing the projections of the data in the new space. **V** contains the *K* main eigenvectors of the covariance matrix arranged in columns and it acts as the basis change matrix. After this process, each image with *N_x_* × *N_y_* pixels has been transformed into a vector with *K* components.

Some authors have applied PCA in mobile robots localization [7,17]. However, this classical formulation of PCA presents two disadvantages. Firstly, the projections cannot be computed incrementally, and secondly, the descriptors obtained are not invariant against changes in the robot orientation. To overcome this second limitation, we make use of the PCA formulation proposed by [18], where the database is created taking into account all the possible orientations of the robot when capturing each reference image.

### 3.3. Histogram of Oriented Gradients

Histogram of Oriented Gradients (HOG) descriptors were first introduced in [19]. The idea behind these descriptors is that the appearance of the objects in a scene and their shape can be described by means of the gradient intensity and direction. The basic implementation consists in dividing the image into small connected regions, named cells, and building a histogram of gradient orientations for each cell. The final descriptor is composed of this set of histograms arranged consecutively in an only vector. These descriptors have been used traditionally in detection of objects in scenes. Other authors have developed improved versions both in computational cost and effectiveness for people detection [20].

The experience with this kind of descriptors in robot mapping and localization is very limited. Hofmeister *et al.* [21] use a weighted histogram of oriented gradients in small mobile robots moving into a small and controlled environment, using very low resolution images and without visual aliasing. The algorithm developed by the authors works well under these restrictive conditions and only if the robot orientation is similar to the orientation of the images in the map. The same authors [22] have developed a comparative study between HOG and other appearance techniques applied to the localization of mobile robots in small environments, with similar results.

Inspired in this description method, we have implemented an HOG descriptor applicable to panoramic images that offers rotational invariance and allows us to compute both the position and the orientation of the robot.

### 3.4. Gist and Prominence of Scenes

*Gist* descriptors try to imitate the human ability to immediately recognize an scene by means of identifying some regions that have a prominent color and/or texture with respect to the rest of the image. This concept was first introduced by Oliva and Torralba [14,23] as *holistic representation of the spatial envelope*. Mathematically, they codify the spatial information through 2D Discrete Fourier Transform computed in several regions equally distributed throughout the image. This set of data is then dimensionally reduced by means of Principal Components Analysis. More recently, the same authors make use of steerable wavelet pyramids instead of Fourier Transforms [24]. The authors show how this kind of descriptors are able to classify image sets according to subjective features such as degree of naturalness, aperture, roughness, *etc*. In this work we give the descriptors a different use as our map is composed of a set of images with similar appearance. Our objective is to build a descriptor based on the *gist* concept but robust against *visual aliasing* and invariant against rotations on the ground plane.

More recent works make use of the, *prominence* concept together with *gist*, which refers to the zones in an image which stand out comparing to their neighbors [25]. This descriptor is build with the information of intensity, orientation and color.

We can find in the literature few applications of this descriptor in mobile robotics. For example, Chang *et al.* [26] present a localization and navigation system based on these *gist* models and Murillo *et al.* [27] make use of *gist* descriptors to solve the localization problem in urban areas. However these two works compute *gist* only in certain areas of the image. As our objective is to test the performance of global appearance descriptors, inspired in [25] we have designed a unique descriptor per scene that, when computed from a panoramic image, presents rotational invariance.

## 4. Implementation of the Global Appearance Descriptors

In this section we detail how we have implemented robust and rotationally invariant descriptors to represent globally the panoramic scenes.

First, in the *learning phase*, to build the map or representation of the environment we compute the descriptors of each image in the dataset.

Then, to carry out the *validation phase*, we study if this map is useful with localization and orientation estimation purposes. We solve the localization problem as an *image retrieval* problem. The robot captures a new image at time *t* from an unknown position, computes its descriptor and compares it with all the descriptors in the map. As a result, we get a distances vector at time *t*: *l⃗_t_* = {*l_t_*_1_, *l_t_*_2_, …, *l_tn_*} = {*l_tj_*} *i* = 1, …, *n* where *l_tj_* = *dist*(*d_t_*(*x*, *y*), *d_j_*(*x*, *y*)). In this work we make use of the Euclidean distance.

Using a sorting algorithm we arrange these distances in ascending order. After that, we retain the closer neighbors. We name *nearest neighbor* the image in the database whose descriptor has minimum distance *l_tj_*, the *second nearest neighbor* the image in the database with the following distance value, and so on. Using this information, with a localization algorithm we can estimate the position of the robot in the topological map.

Once the position has been computed, the next step consists in estimating the orientation of the robot. With this aim, we compare the descriptor of the image captured at time *t* and compare it with the *nearest neighbor*.

In the following sections we detail these steps for the four description methods compared.

### 4.1. DFT Descriptor

The map is composed of a set of descriptors. Each descriptor is represented by two matrices: the modules *A_i_*(*u*, *y*), with size *N_x_* × *k*_1_, and a phase matrix Φ*_i_*(*u*, *y*), with size *N_x_* × *k*_1_.

First, we use the modules matrix to estimate the position of the robot. We compute the distance between the modules matrix *A_t_*(*x*, *y*) of the image currently captured by the robot and the modules matrices in the database *A_i_*(*x*, *y*), *i* = 1, …, *n*. As a result, we retain the *nearest neighbors* from the map and we use this information to estimate the position of the robot.

Once the position has been computed, we estimate its orientation, using the argument matrix Φ*_t_*(*u*, *y*) computed for the currently captured image and the argument matrix of the nearest *nearest neighbor* image in the database. We compare these two argument matrices taking profit of the Fourier shift theorem (Equation (2)) and as a result, the relative orientation can be estimated.

The Fourier Signature parameter we will try to optimize with the experiments is the number of columns retained from the signature, *k*_1_ to arrive to a compromise between the computational cost and the accuracy during the localization process.

### 4.2. PCA Descriptor

The PCA descriptor we use is described in the works of Jogan *et al.* [18]. This model uses the specific properties of panoramic images to create a set of *N* spinning images from each original panoramic image, so we get *N* data vectors per original image (Figure la). After that, these data vectors are compressed by means of PCA. If this compression step was carried out with the traditional PCA approach, the computational cost would make it unbearable to be carried out in real time. However, in this case, the inner product C = **X***^T^* · **X** is composed of *n* × *n* circulant blocks whose size is *N* × *N* (*n* is the number of locations). We can take profit of this property to reduce the computational cost by transforming the problem of solving the SVD of C to the problem of solving *N* decompositions of order *n*.

**Figure 1.**
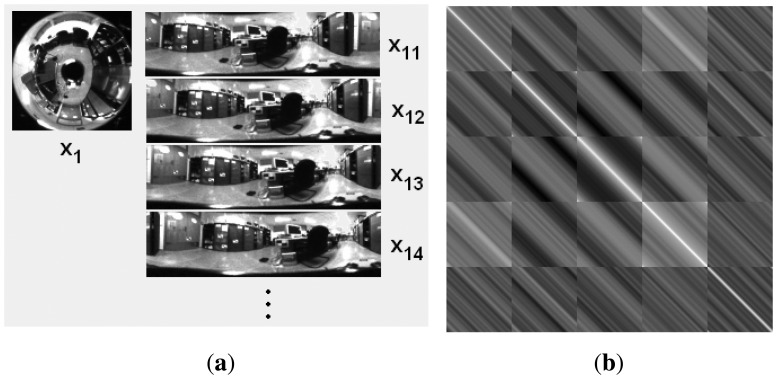
**(a)** Omnidirectional image (*x*_1_), corresponding panoramic image (*x*_11_) and some samples of artificially rotated versions (*x*_12_, *x*_13_, *x*_14_, …), to carry out PCA with rotated images (**b**) Inner product matrix **Q** corresponding to a set of images from *P* = 5 locations and *N* = 128 rotations per location.

Figure 1a shows the different versions of a panoramic scene artificially generated to build the data matrix to perform PCA analysis with rotations and (b) the inner product C corresponding to a set of images from *n* = 5 locations and *N* = 128 rotations per location. We can express then:

(3) $$Q=X^{T}\cdot X=\left(\begin{array}{cccc} Q_{11} & Q_{12} & \cdots & Q_{1n}\\ Q_{21} & Q_{22} & \cdots & Q_{2n}\\ \vdots & \vdots & \vdots & \vdots \\ Q_{n1} & Q_{n2} & \cdots & Q_{nn} \end{array}\right)$$

where *Q_ij_* are circulant matrices with size *N* × *N*. The eigenvectors of all those circulant matrices are the same, independently on the values in the matrices. This set of vectors is: $F=[{\vec{\upsilon }}_{0}^{\prime },{\vec{\upsilon }}_{1}^{\prime },\cdots {\vec{\upsilon }}_{N-1}^{\prime }]$ where ${\vec{\upsilon }}_{k}^{\prime }={\left[1,\omega^{k},\omega^{2\cdot k},\ldots ,\omega^{\left(N-1\right)\cdot k}\right]}^{T}$, *k* = 0, …, *N* − 1, where $\omega =e^{\frac{-2\pi j}{N}}$, $j=\sqrt{-1}$. On the other hand, the eigenvalues can be computed as $\lambda_{k}^{\prime }=\sum_{l=0}^{N-1}q_{l}\cdot e^{\frac{-2\pi jlk}{N}}$, where {*q_l_*} = [*q*_0_, *q*_1_, …, *q_N_*_−1_] is the first row of each block *Q_ij_*.

Since all the *Q_ij_* blocks present the same set of eigenvectors ${\vec{\upsilon }}_{k}^{\prime }$, we can state the problem of diagonalizing **Q** as:

(4) $$Q\cdot {\vec{\omega }}^{\prime }=\mu \cdot {\vec{\omega }}^{\prime }$$

where the eigenvectors present the form:

(5) $$\begin{array}{ll} {\vec{\omega }}_{k}^{\prime }={\left[\alpha_{k1}\cdot {\vec{\upsilon }}_{k}^{\prime T},\alpha_{k2}\cdot {\vec{\upsilon }}_{k}^{\prime T}\ldots \alpha_{kn}\cdot {\vec{\upsilon }}_{k}^{\prime T}\right]}^{T}, & \text{with}\;k=1,\ldots ,N \end{array}$$

Equation (4) can be rewritten as:

(6) $$\begin{array}{ll} \overset{n}{\underset{j=1}{\text{∑}}}Q_{ij}(\alpha_{kj}{\vec{\upsilon }}_{k}^{\prime })=\mu \alpha_{ki}{\vec{\upsilon }}_{k}^{\prime }, & \text{with}\;i=1,\ldots ,n \end{array}$$

Also, since ${\vec{\upsilon }}_{k}^{\prime }$ is an eigenvector for every block *Q_ij_*, Equation (6) can be simplified:

(7) $$\begin{array}{ll} \overset{n}{\underset{j=1}{\text{∑}}}\alpha_{ij}\lambda_{ij}^{\prime k}{\vec{\upsilon }}_{k}^{\prime }=\mu \alpha_{ki}{\vec{\upsilon }}_{k}^{\prime }, & \text{with}\;i=1,\ldots ,n \end{array}$$

where $\lambda_{ij}^{\prime k}$ is an eigenvalue for *Q_ij_* corresponding to the eigenvector ${\vec{\upsilon }}_{k}^{\prime }$. It implies a new eigendecomposition problem:

(8) $$\Lambda \alpha_{k}=\mu \alpha_{k}$$

where:

(9) $$\begin{array}{lll} \Lambda =\left(\begin{array}{cccc} \lambda_{11}^{\prime k} & \lambda_{12}^{\prime k} & \cdots & \lambda_{1n}^{\prime k}\\ \lambda_{21}^{\prime k} & \lambda_{22}^{\prime k} & \cdots & \lambda_{2n}^{\prime k}\\ \vdots & \vdots & \vdots & \vdots \\ \lambda_{n1}^{\prime k} & \lambda_{n2}^{\prime k} & \cdots & \lambda_{nn}^{\prime k} \end{array}\right) & \text{and} & {\vec{\alpha }}_{k}={\left(\begin{array}{cccc} \alpha_{k1} & \alpha_{k2} & \cdots & \alpha_{kn} \end{array}\right)}^{T} \end{array}$$

Since **Q** is symmetric by blocks, **Λ** is also symmetric, so we have *n* independent eigenvectasors *α⃗_k_* which provide us with *n* eigenvectors ${\vec{w}}_{k}^{\prime }$ in Equation (5). If we apply this method for each ${\vec{\upsilon }}_{k}^{\prime }$, we will obtain *N* · *n* lineally independent eigenvectors for **Q**.

Thanks to this procedure, the problem of computing the SVD decomposition of **Q** (with size *N* · *n*) can be decomposed in *N* problems with size *n*, with a substantially lower computational cost.

**Figure 2.**
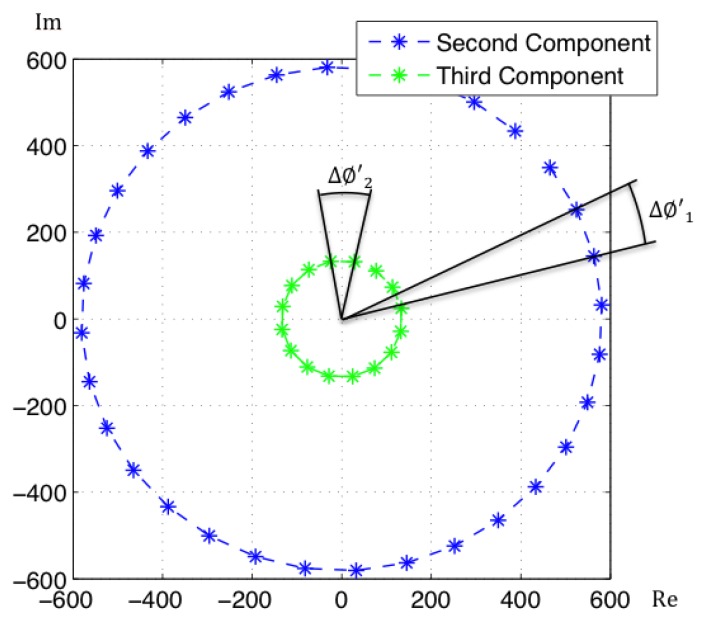
Graphical representation in the complex plane of two components of the projections of a 32 rotations set for an image.

Since the projection basis is complex, so will be the coefficients in the projections of the images. It can be proved that the coefficients of an image and its rotated versions have the same modulus, with only a change in argument [18]. Moreover, the phase lag between the coefficients of two consecutive rotated versions of an image is constant as Figure 2 shows. This phase lag can be calculated as:

(10) $$\Delta \phi_{j}=\arctan\frac{\text{Re}(q_{(i+1)j}-q_{ij})}{\text{Im}(q_{(i+1)j}-q_{ij})}$$

where *q_ij_* are the coefficients of the projections of all the images and their rotated siblings. *i* = 0, …, *N* is the rotation number and *j* = 1, …, *K* is the number of coefficient. This way, knowing the angle between the coefficients of the original panoramic image and the first rotation, the rest of coefficients can be artificially placed in the complex plane. Hence, the localization of the robot in the map can be addressed in two steps: first the localization of the robot in the database is carried out by comparison of the coefficients modules and second, the estimation of the phase is carried out simulating the projections of all the rotations. The angular resolution depends on the number of rotated siblings for each image included in the database: min (*θ*) = 2 · *π*/*N*.

To conclude, in the case of Rotational PCA, the database is made up of the projections (or descriptors) of each scene, arranged in a matrix **P** with size *K* × *n* (*K* is the number of main eigenvectors retained and *n* is the number of locations in the database), the basis change matrix **V** with size *K* × *N_x_* · *N_y_* and the *K* phase lags between components of the projections of each image and its first rotated sibling.

The localization process is carried out by projecting the input image at time *t* onto the new eigenspace, to get the descriptor *p⃗_t_*. The localization is estimated by computing the module of the descriptor and comparing with the module of the descriptors in the database to obtain the nearest neighbors. Then, the orientation is estimated using the information in the phase lags stored in the map.

### 4.3. HOG Descriptor

**Figure 3.**
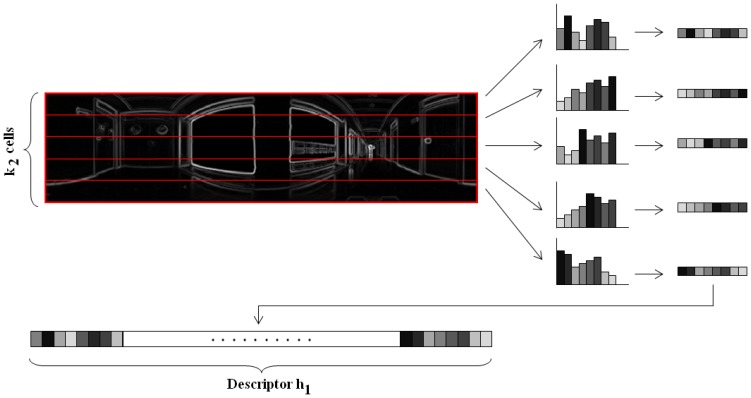
Distribution of horizontal cells on a panoramic image to build a rotationally invariant descriptor *h⃗*_1_.

**Figure 4.**
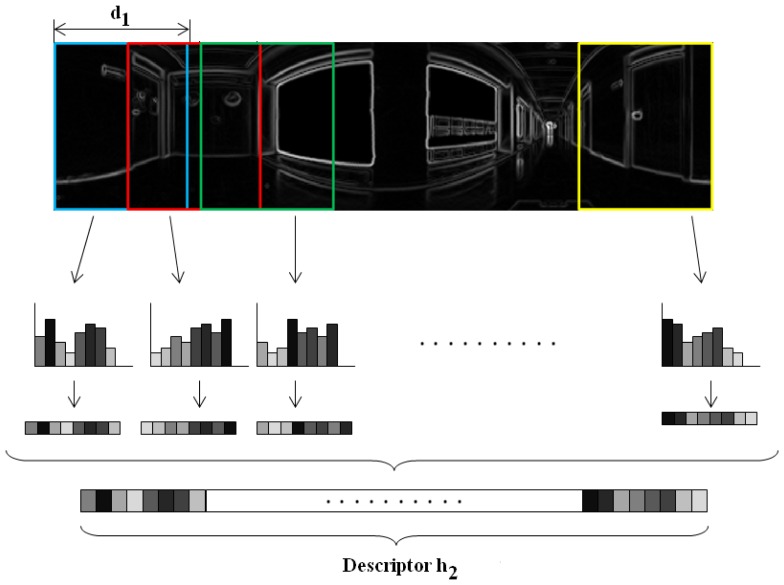
Distribution of overlapping vertical cells on a panoramic image to build a descriptor *h⃗*_2_ that permits estimating the orientation of the robot.

These are the steps we follow to build the descriptor:

1. *Calculating the gradient of each scene*. We convolve with two masks, *D_x_* = [−1 0 1] and *D_y_* = [−1 0 1]*^T^* to extract the horizontal *i_x_* = i*D_x_ and the vertical *i_y_* = i*D_y_ components. After this step we compute the gradient magnitude $|G|=\sqrt{i_{x}^{2}+i_{y}^{2}}$ and its orientation $\Theta =\arctan\frac{i_{y}}{i_{x}}$. Both |*G*| and Θ are *N_x_* × *N_y_* matrices.
2. *Orientation binning*. The image is divided in cells and the histogram from each cell is computed. We have decided to work with 8 histogram bins uniformly distributed between 0 and 180 degrees. Each pixel in the cell contributes to the histogram bin that contains the orientation of that pixel *θ*, with a weighting factor equal to the gradient magnitude of the pixel |*G*|. In this point, we introduce a change respect to the classical HOG descriptor. We have decided to build these histograms twice, first dividing the panoramic scene into horizontal cells, to get a rotationally invariant descriptor *h⃗*_1_, as Figure 3 shows and then, dividing the scene into vertical cells with overlapping, to build a second descriptor *h⃗*_2_ that allows us to estimate the robot orientation with precision, as shown on Figure 4. This is one of the contributions of the paper.
3. *Normalization*. The two descriptors are now normalized to make them robust against changes in lighting conditions and contrast of the scenes. We group the cells into larger blocks spatially connected. The HOG descriptors will be constituted by the cell histograms normalized using the information in the blocks that contain each cell. We have decided to use rectangular blocks containing 3 cells with 1 cell overlapping to normalize the blocks.

The variable parameters of the HOG descriptor are the number of horizontal cells *k*_2_, the number of vertical cells *k*_3_ and the width of vertical cells *d*_1_. As a result we get two descriptors, the first one will be used with localization purposes and the second one to estimate the robot orientation. Once built the HOG descriptors, then localization is estimated by calculating the minimum distance between the *h⃗*_1_ descriptor in the database an the current image. The orientation is obtained by successive rotation and comparison of the *h⃗*_2_ vector of the input image at time *t* and the nearest image in the map. The angle accuracy we are able to detect between two shifted images is proportional to the distance between consecutive vertical cells.

### 4.4. Gist Descriptor

**Figure 5.**
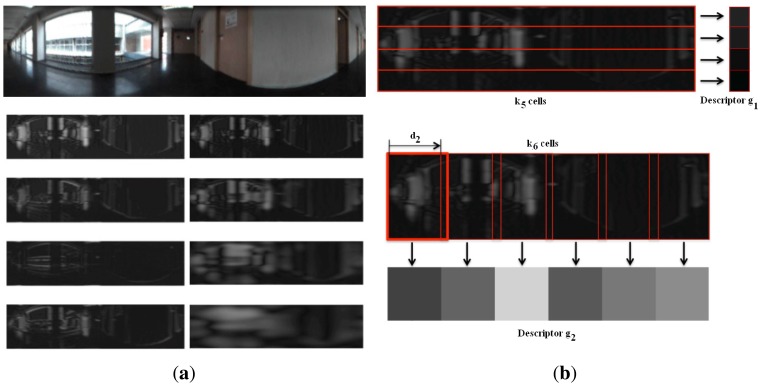
**(a)** Sample image filtered with *k*_4_ = 4 Gabor filters with {0, 45, 90, 135}*deg* orientation in 2 scales and (**b**) extraction of the values to build the two descriptors from each filtered image with horizontal and vertical cells.

Our *gist* descriptor is built following these steps:

1. *Building an image pyramid*. The objective is to describe image properties at different scales and between scales. The first level is the original image. To obtain every new level, we apply a Gaussian low pass filter and the image is subsampled to obtain a new image with size 0.5*N_x_* × 0.5*N_y_*.
2. *Gabor filtering*. To include orientation information, each level of the pyramid is filtered with a bank of Gabor filters with *k*_4_ orientations uniformly distributed between 0 and 180 degrees. As a result we get *k*_4_ matrices per pyramid level with information on the analyzed directions. We apply it to the two first images of the pyramid so we get 2 · *k*_4_ resulting matrices.
3. *Blockification*. To reduce the amount of information, we group the pixels of every resulting matrix in blocks by means of computing the average intensity value that have the pixels in each block. Usually, a set of square blocks is defined on the image to carry out the blockification process [25]. However, we have decided to make the block division in a similar fashion as in HOG: first we compute a descriptor with horizontal blocks (to be used with localization purposes) and then a second descriptor with overlapping vertical blocks (to compute the orientation), as shown on Figure 5. This blockification process is a contribution of our work and it provides us with a rotationally invariant *gist* descriptor.

Once built the descriptor, the localization and orientation estimations are carried out using respectively the horizontal blocks descriptor and the vertical blocks descriptor, using the same procedure as in HOG.

The configurable parameters of the *gist* descriptor are then (a) the number of Gabor masks, *k*_4_ (b) the number of horizontal cells *k*_5_, (c) the number of vertical cells *k*_6_ and (d) the width of vertical cells *d*_2_.

### 4.5. Removal of the Effects of Changing Lighting Conditions

When a robot has to move autonomously in a real environment using vision as input data, it has to cope with the problem of changing lighting conditions. These conditions may vary considerably depending on the moment of the day and of the year and on the use of natural or artificial illumination. These changes will introduce perceptible changes in the appearance of the scenes.

After several works ([28,29]), we have decided to make use of homomorphic filtering techniques [30] on the panoramic images as a preprocessing step before building the descriptors. The homomorphic filtering allows us to filter separately the luminance and reflectance components of an image. Thus, we can control the influence of each component on the image appearance. The separation of these components can be done with the natural logarithm:

(11) $$\begin{array}{r} i(x,y)=l(x,y)\times r(x,y)\\ z(x,y)=\ln(i(x,y))=\ln(l(x,y))+\ln(r(x,y)) \end{array}$$

where *i*(*x*, *y*) is the panoramic image, which can be expressed as the product of the luminance *l*(*x,y*) and the reflectance components *r*(*x*, *y*). After separating these components, we apply a high pass filter on the frequency domain, due to the fact that the low frequency components are associated with the lighting conditions of the scene and the high frequency ones with the reflectance, thus, a high pass filter (built from a Butterworth filter) is expected to reduce the effects of changing lighting conditions.

(12) $$\begin{array}{c} F[\{z(x,y)\}]=F[\left\{\ln(l(x,y))\right\}]+F[\{\ln(r(x,y))\}]\\ F[\{z^{\prime }(x,y)\}]=F[\{z(x,y)\}]\cdot H(u,\upsilon ) \end{array}$$

where *H*(*u*, *υ*) is the high pass filter transfer function in the frequency domain and *F* is the 2D-DFT operator.

## 5. Experiments and Results

In this section, we compare the performance of the four global appearance descriptors in the tasks of map creation and localization. For these purposes, we make use of two different images databases captured in different environments under realistic lighting conditions. We have carried out four different experiments with this goal. First, we evaluate the computational cost of building the representations of the database. We evaluate the necessary time and memory depending on the value of the most relevant parameters of the descriptors. Second, we test the performance of the descriptors to solve the global localization task as an image recovery problem. After that, we test the robustness of the descriptors to solve the same task, but when occlusions, noise or changes in lighting conditions are present. To end, we study the behavior of the descriptors to solve a probabilistic localization task, using the Monte Carlo algorithm.

In this section we first introduce the images's databases we have used to carry out the experiments and then we present the results of the four experiments.

### 5.1. Images' Databases

**Figure 6.**
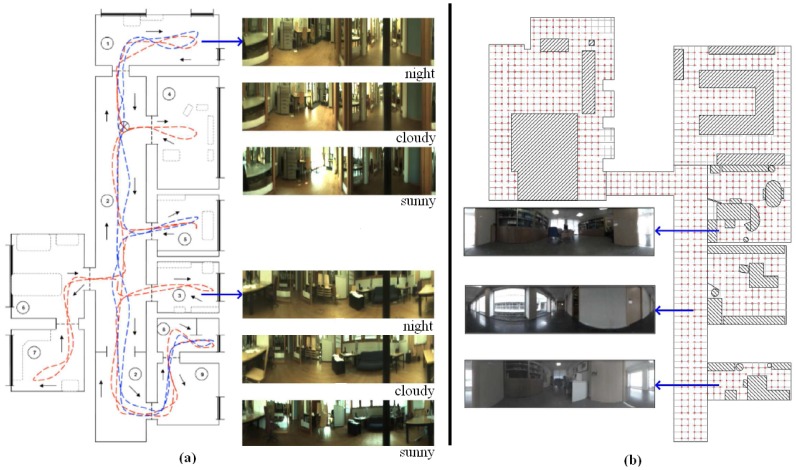
Bird eye's view of (**a**) the COLD and (**b**) the *Quorum* databases and some sample panoramic images extracted from them.

We make use of two databases, captured with two different catadioptric systems (with different geometry). This fact does not affect the process to compute the descriptors since we approach the problem from a topological point of view. Therefore, a camera calibration process is not necessary. First, the *Quorum* database has been captured by ourselves in an indoors environment (Quorum 5 building, at Miguel Hernandez University, Spain). This database includes a corridor and some offices and meeting rooms. This database is composed of two sets of images. The first one (training set) is composed of 873 panoramic 128 × 512 images which have been captured on a dense 40 × 40 cm grid of points. The second one (test set) is composed of 546 images captured in all the rooms, in some half-way points among the grid points and with different orientations and times of day. Figure 6 shows a bird eye's view of the grid points where the robot captured the training set and some samples of panoramic scenes.

The second database, named COLD, has been captured by a third party [31]. It consists on three sets of about 4,500 omnidirectional images each. We have transformed them to 128 × 512 panoramic images. They were captured along a route that the robot traversed, visiting several rooms connected by a corridor. This route was repeated three times under different lighting conditions (*sunny*, *cloudy* and *night*), that is why this database is composed of three sets of images. Figure 6 shows a bird eye's view of the route traversed by the robot when capturing this database and some samples of panoramic scenes, extracted from each one of the three available sets.

### 5.2. Building the Visual Memory

The objective of this section is to compare the performance of the four descriptors during the task of creating a representation or map of the environment using the two images databases. We will show some results about the computational cost to build the map and the necessary memory to store it, depending on the value of the descriptors' parameters. In the following subsections we will make some additional experiments to test the utility of these representations in a localization task. After all the experiments, we will have the necessary information to know which is the best descriptor and the optimal parameters to arrive to a compromise between computational cost and accuracy in localization.

**Figure 7.**
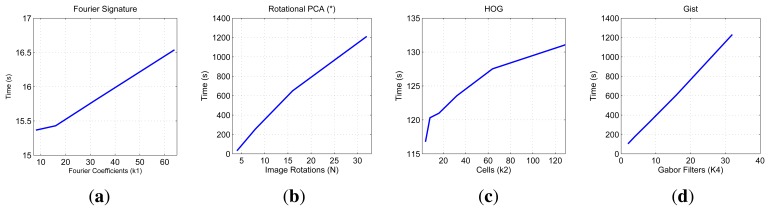
COLD database. Necessary time to compute the representation (map) of the environment.

**Figure 8.**
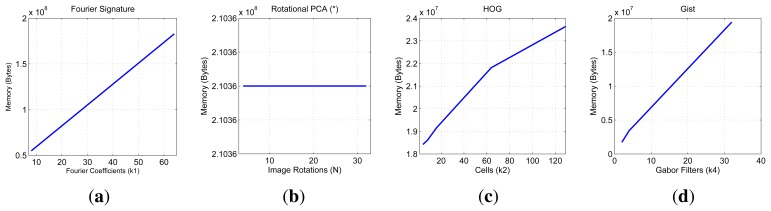
COLD database. Necessary memory to store the database.

First, we show on Figure 7 the necessary time to compute all the descriptors and on Figure 8 the necessary memory to store them when we use one of the sets of the COLD database, depending on the main parameters of the description methods.

First, the main parameter of the Fourier Signature is the number of columns *k*_1_ we retain to compose the descriptor. This descriptor is composed of a module matrix and a phase matrix, both with a size *N_x_* × *k*_1_. From the figure we deduct that both the memory and time proportionally increase as we select more columns. Anyway, the increase in time is not significant because the cost of computing the DFT of each row is the same independently of *k*_1_, and the only difference is computing the module and phase of more or less components.

In the case of Rotational PCA, the database is made up of the projections (or descriptors) of each scene, arranged in a matrix **P** with size *K* × *n* (*K* is the number of main eigenvectors retained and *n* is the number of locations in the database), the basis change matrix **V** with size *K* × *N_x_* · *N_y_* and the *K* phase lags between components of the projections of each image and its first rotated sibling. In this case the main parameter we have considered is the number of artificial image rotations *N* when creating the database. We have left *K* constant and equal to the maximum number of eigenvectors available. The figures show how *N* does not affect the necessary memory (since we have to store just the matrices **P** and **V**, whose size does not depend on *N*). However, in the case of the time, there is a substantial increase as *N* does. Due to this increase in computational cost and the memory requirements during the process, we have not been able to test this algorithm with the whole database. All the results are shown for a database size of 200 images. That is the reason why an asterisk is shown in all the PCA figures. This way, these results are not comparable to the rest of descriptors.

As for HOG, the parameter we have varied to shown these graphics is the number of horizontal cells, *k*_2_ and in the case of *gist*, we have studied the influence of the number of Gabor masks *k*_4_, since in previous works we have shown how these are the parameters which have a greater influence in the behavior of the descriptor [32]. In both cases, the memory increases with the number of cells and masks, however in all cases it is one magnitude order lower comparing to Fourier Signature. The time also increases and we can see how *gist* is the computationally more expensive process and Fourier Signature is the less expensive one.

Comparatively, PCA is the computationally heaviest method, despite of using the properties of the circulant matrices to carry out the SVD decomposition. Fourier Signature is the fastest algorithm and *gist* is the most compact descriptor, thanks to the blockification method used to compress the information.

### 5.3. Image Recovering and Orientation Estimation

In this section we test the utility of the map created in the previous section to solve the global localization task. The robot has no information about its position at time *t* so we solve the localization problem as an image recovering task.

**Figure 9.**
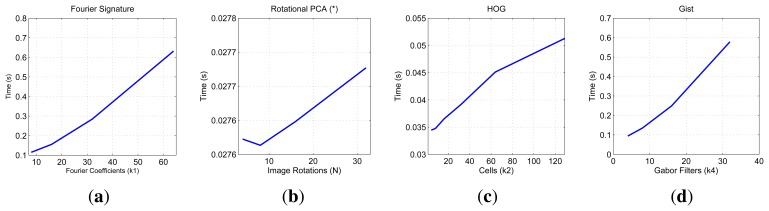
COLD database. Computational cost of the localization process.

First we show on Figure 9 the computational cost to compute the descriptor of the image, compare it with the rest of images in the database and estimate both the position and the orientation of the robot. These data have been obtained using one set of the COLD database. This a relevant information as it shows whether each method is able to work in a real time application.

We must remind that the results of Rotational PCA are given for a reduced version of the database with only 200 images. The PCA curve shows how the computational cost of the localization process is quite stable (the range shown at the y-axis is very short). This is due to the fact that the size of the descriptor is *K* and it does not depend on *N* thus the computational cost to compare one descriptor to all the descriptors in the model is constant. Comparing the rest of descriptors, *gist* and FS present a similar computational cost and HOG has the lowest cost. Anyway, depending on the value of the parameters, the three descriptors allow us to make the robot localization in real time in a large database.

We carry out the image recovering experiment (localization) using both databases. In the *Quorum* database we use the intermediate images as test images, and the map is composed of the grid images. In the COLD database, all images are used as test images. When a new image is tested, it is removed from the map and compared to the remaining images.

We express the result of the image recovering experiments by means of *Recall* and *Precision* curves. Each curve shows the evolution of the experiment as we carry out the image retrieval with each one of the test images. The *recall* indicates the number of images correctly classified regarding the total number of test images, and the *precision* indicates the total number of images correctly classified with respect to the number of images tested so far. The most important data of this type of graphs is the final point as it shows the most general result of the experiments. The final precision is the percentage of correctly classified test images.

**Figure 10.**
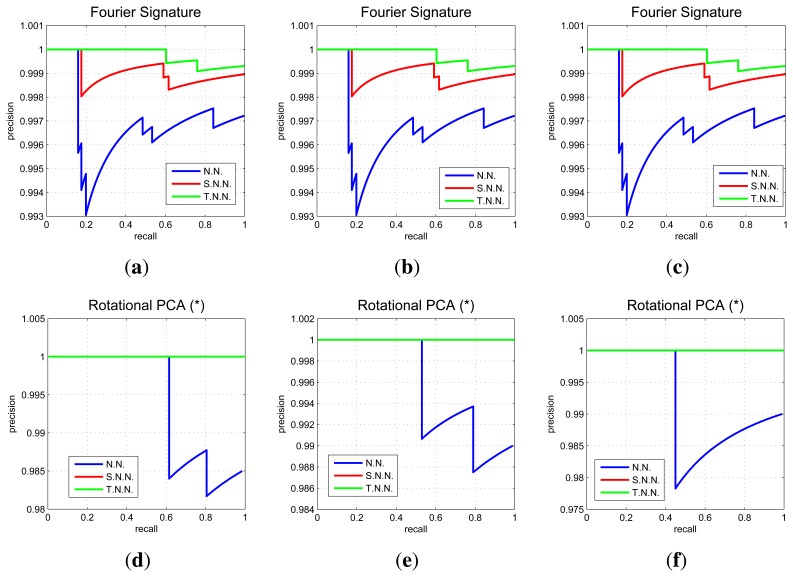

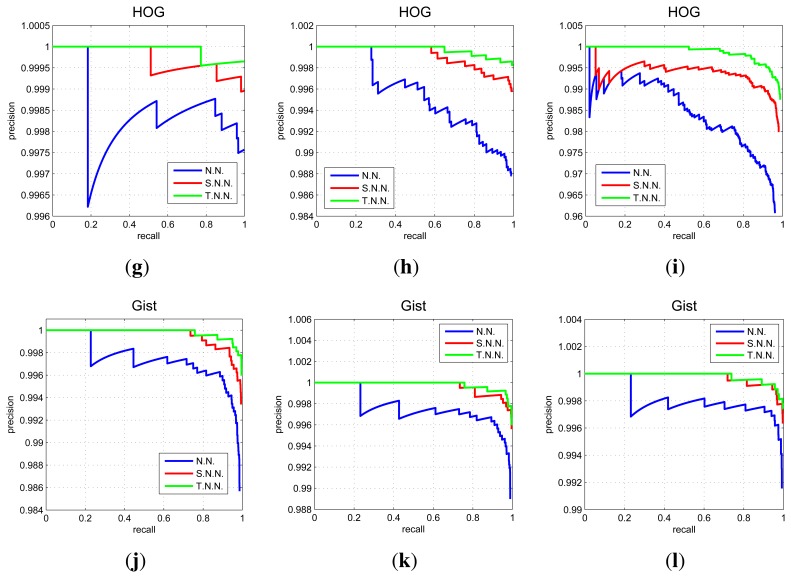
COLD database. Recall and precision curves for FS and *k*_1_ = (**a**) 8, (**b**) 16, (**c**) 32 components, PCA with rotations and *N* = (**d**) 8, (**e**) 16, (**f**) 32 rotations, HOG and *k*_2_ = (**g**) 4, (**h**) 16, (**i**) 64 cells and *gist* and *k*_4_ = (**j**) 4, (**k**) 16, (**l**) 64.

In Figure 10 we show some relevant graphs obtained using the COLD database. Each graph shows three recall and precision curves. To draw the first curve (*NN*) we consider that a test image is correctly classified if the nearest neighbor in the map is closer than 10 cm to the point where the test image was captured. The second curve (*SNN*) is drawn considering a correct classification when the nearest neighbor or the second nearest neighbor is within this threshold, and the third curve (*TNN*) considers a correct classification when the first or the second or the third nearest neighbor is within this 10 cm threshold. This way, the first curve is the most restrictive one. The second and the third curves also consider a good classification when the second or the third neighbor are geometrically close to the test images.

**Figure 11.**
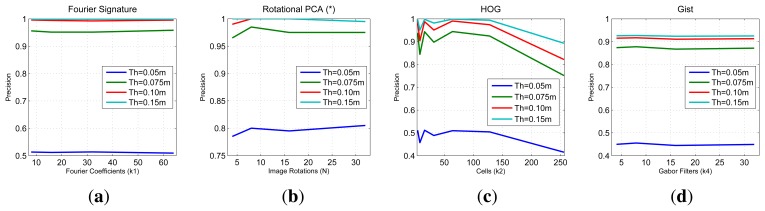
COLD database. Final precision results (expressed in parts per unit) depending on the parameter of each descriptor (**a**) Fourier Signature (**b**) rotational PCA (**c**) HOG and (**d**) *gist*.

In Figure 11 we sum up the information from all the image retrieval experiments we have carried out. These charts present the final result of precision (expressed in parts per unit) for each experiment *versus* the main parameters of the descriptors. To analyze a generic situation, we have defined four different geometric thresholds around the point where the test image was captured. In general, FS and *gist* present a constant precision independently on the parameter of the descriptor, although FS presents a slightly better behavior. HOG presents as good localization results as FS when we use an intermediate number of cells (*k*_2_). PCA presents the best localization results (when using a limited database of 200 images).

**Figure 12.**
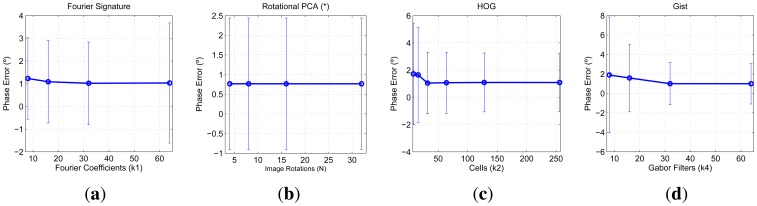
COLD database. Mean orientation error when comparing each test descriptor with its nearest neighbor in the database depending on the parameter of the descriptor (**a**) Fourier Signature; (**b**) rotational PCA; (**c**) HOG and (**d**) *gist*.

To finish this experiment, we are also interested in testing the performance of the descriptors when estimating the relative orientation between the test image and the retrieved image from the database. These data are shown in Figure 12. These charts show the mean and variance of the error when computing this relative orientation for all the test images. The relative orientation is computed comparing the descriptor of each test image and the nearest neighbor in the database. PCA with rotations offers the best results, but when using the other three descriptors, these errors can be limited to about 1 degree if the parameters are correctly tuned. In the case of PCA with rotations, the mean orientation error is constant independently on N, due to the fact that, once the robot is well localized, we can interpolate between consecutive rotations since we know the phase lag between consecutive projections.

### 5.4. Robust Localization

In this section we test the performance of the descriptors in a localization task under some typical situations: different lighting conditions, occlusions and noise.

**Figure 13.**
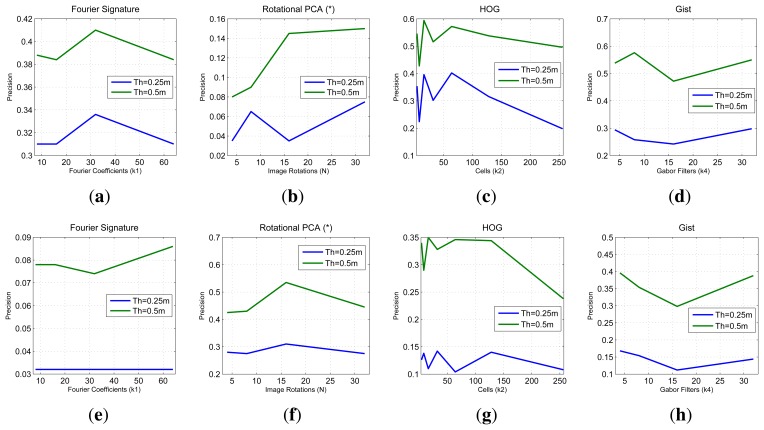
COLD database. Precision in localization using the *sunny* images as map database and (**a**), (**b**),(**c**), (**d**) cloudy images and (**e**), (**f**), (**g**), (**h**) night images as test.

In the first experiment we make use of the COLD database. We have taken the images in the *sunny* set as a reference (map) and we take as test images those in the *cloudy* and in the *night* sets. Figure 13 shows the precision (expressed in parts per unit) in this experiment. This figure shows how the precision in localization decreases when there is a change in the lighting conditions. Comparing the figures we could state that *gist* and HOG are the more robust descriptors against changes in lighting conditions.

**Figure 14.**
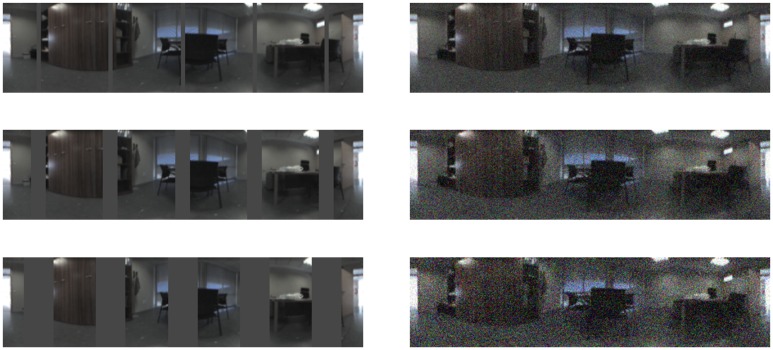
Some examples of test images with different artificial occlusion percentage and with added Gaussian noise with different variances.

The next experiment has been carried out with the *Quorum* database. It shows the influence of occlusions and noise. We have artificially added some percentage of occlusions to the test images before computing their descriptors or Gaussian noise with some variance. Figure 14 shows some sample images from the *Quorum* database with added occlusions or noise. Table 1 sums up the precision results (expressed as a percentage) after carrying out the localization process with all the test images, depending on the percentage of occlusion and noise variance. To get these data we have considered a correct match when the nearest neighbor is one of the four images around the test image (as the map images have been captured on a regular grid).

As far as occlusions are concerned, the precision clearly decreases when the percentage of occlusions increases. However, HOG and *gist* present the most stable behavior. In the presence of noise, PCA with rotations is able to cope with it as there is no appreciable change in precision. Fourier Signature also presents good results against Gaussian noise.

To conclude this subsection, the results of global localization that we have obtained show how the behavior of all the descriptors gets worse when noise, occlusions or changes in lighting conditions are present. HOG and *gist* are able to cope better with changing lighting conditions and partial occlusions, and PCA with rotations and Fourier Signature are able to avoid the effects of noise. Anyway, in real applications it is usual that a probabilistic approach is used to estimate the position and orientation of the robot. In this cases, the initial global localization of the robot is refined with additional data. To conclude the experimental section, in the next subsection we show the performance of the descriptors to solve a probabilistic localization task. Due to its high computational cost when working with large databases, we have discarded PCA with rotations and we compare the performance of Fourier Signature, HOG and *gist*.

### 5.5. Monte Carlo Localization

Once we have carried out the global localization experiments, we are interested in testing the performance of the descriptors in a probabilistic localization task. In this section we present the formulation of the Monte Carlo algorithm we have implemented with this aim. In this problem we not only take into account the current observation but also all the data available till this moment: we try to estimate the robot's position and orientation *x_t_* = (x, y, *θ*)) at time *t* using the set of previous and the current image descriptors *d*_1:_*_t_* = {*d*_1_, *d*_2_, …, *d_t_*} and the movements *u*_1:_*_t_* = {*u*_1_, *u*_2_, …, *u_t_*} of the robot. We consider the robot makes the movement *u_t_* from time *t* − 1 to time *t* and then it captures a new image and computes its descriptor *d_t_*.

We have previously built a map of the environment where the robot moves, which is composed of a set of *n* landmarks *L* = {*l*_1_, *l*_2_, …, *l_n_*} which position is known. These landmarks form a grid in the environment. Each landmark *l_j_* is represented by the descriptor *d_j_* that describes the global appearance of the omnidirectional image captured from each position, thus *l_j_* = {(*l_j_*_,x_, *l_j_*_,y_), *d_j_*}.

To test the performance of the descriptors, we have decided to state this problem in a probabilistic fashion: we will estimate a probability function *p*(*x_t_*∣*z*_1:_*_t_*, *u*_1:_*_t_*) over the space of all possible poses, conditioned on all the data available until time *t*, the observations *d*_1:_*_t_*, movements performed *u*_1:_*_t_* and the map. With this aim, we follow the principles of the Monte Carlo localization method to represent the probability density function *p*(*x_t_*∣*z*_1:_*_t_*, *u*_1:_*_t_*) as a set of *M* weighted random samples $\left(\chi_{t}=\{x_{t}^{i},i=1\ldots M\}\right)$ extracted from it, named particles. Each particle can be understood as a hypothesis of the true state of the robot $x_{t}^{i}=({\text{x}}^{i},{\text{y}}^{i},\theta^{i})$. These algorithms, also named particle filters, have been extensively used in robot localization and SLAM tasks (e.g., [33,34]), due to their efficiency.

The initial set of particles represents the initial knowledge *p*(*x*_0_) about the state of the mobile robot on the map. If we have no information about the initial position of the robot, the initial belief is a set of poses drawn according to a uniform distribution over the robot's map. If the initial pose is partially known up to some small margin of error (local localization or tracking), the initial belief is represented by a set of samples drawn from a narrow Gaussian centered at the known starting pose of the mobile robot. From this initial belief, the *Monte Carlo Localization algorithm* recursively runs these two phases:

**Prediction Phase**: At time *t* a set of particles $\bar{\chi_{t}}$ is generated based on the set of particles *χ_t_*_−1_ and the movement *u_t_*. This step uses the motion model *p*(*x_t_*∣*x_t_*_−1_, *u_t_*), built from the odometry data in our case. As a result, the new set of particles $\bar{\chi_{t}}$ represents the density *p*(*x_t_*∣*z*_1:_*_t_*_−1_, *u*_1:_*_t_*).
**Update Phase**: The image descriptor *z_t_* is used to compute a weight $w_{t}^{i}$ for each particle in the set $\bar{\chi_{t}}$. This weight represents the observation model *p*(*z_t_*∣*x_t_*) and is computed as $\omega_{t}^{i}=p(z_{t}|x_{t}^{i})$. The weights are normalized so that $\text{∑}\omega_{t}^{i}=1$. As a result, a set of particles accompanied by a weight $\bar{\chi_{t}}=\left\{x_{t}^{i},\omega_{t}^{i}\right\}$ are obtained. The resulting set *χ_t_* is calculated by resampling with replacement from the set *χ̄_t_*, where the probability of resampling each particle is proportional to its importance weight $\omega_{t}^{i}$, in accordance with the literature on the SIR algorithm (Sampling Importance Resampling) ([35]). Finally, the distribution *p*(*x_t_*∣*z*_1:_*_t_*, *u*_1:_*_t_*) is represented by the set *χ_t_*.

By means of computing a weight *w^i^* for each particle and performing a resampling process, the Monte Carlo algorithm introduces the current observation *d_t_* of the robot. This step is critical so that this probabilistic process provides us with good results. To compute these weights we compare the descriptor *d_t_* with the rest of descriptors *d_j_*, *j* = 1…*n* and we find the *B* landmarks in the map that are closest in appearance with the current descriptor *d_t_*. We allow the correspondence with several landmarks in the map. From the results obtained in previous works [29], we have decided to compute the weights $\omega_{t}^{i}=p(z_{t}|x_{t}^{i})$, according to Equation (13), which implements a sum of Gaussians centered on each image landmark, considering the difference between descriptors.

(13) $$\omega_{t}^{i}=\sum_{j=1}^{B}\exp\{-\upsilon_{j}{\text{∑}}_{t}^{-1}\upsilon_{j}^{T}\}\exp\{-h_{j}{\text{∑}}_{d}^{-1}h_{j}^{T}\}$$

where, *υ_j_* = (*l_j_*_,x_, *l_j_*_,y_) − (x*^i^*, y*^i^*) is the difference between the position of the landmark *l_j_* and the position (x*^i^*,y*^j^*) of the particle *i*. The matrix Σ*_l_* is a diagonal matrix ${\text{∑}}_{l}=diag(\sigma_{l}^{2},\sigma_{l}^{2})$. The variance $\sigma_{l}^{2}$ is chosen experimentally in order to minimize the error in the localization. *h_j_* = |*d_j_* − *d_t_*| defines the difference between the descriptor associated to the current image observed and the descriptor associated to the landmark *l_j_*. The descriptors are normalized so that the summation of the Euclidean distance of the current descriptor *d_t_* to the rest of the *B* associations equals one, ${\text{∑}}_{j=1}^{B}h_{j}=1$. The matrix ${\text{∑}}_{d}=diag(\sigma_{d}^{2})$ is an *k* × *k* matrix, being *k* the length of the descriptor. In this case, the observation model *p*(*z_t_*∣*x_t_*) is not Gaussian, since it is formed by a sum of Gaussians, being thus multi-modal. This fact generally gives higher weights to particles situated near a landmark that is close in appearance to the current observation.

**Figure 15.**
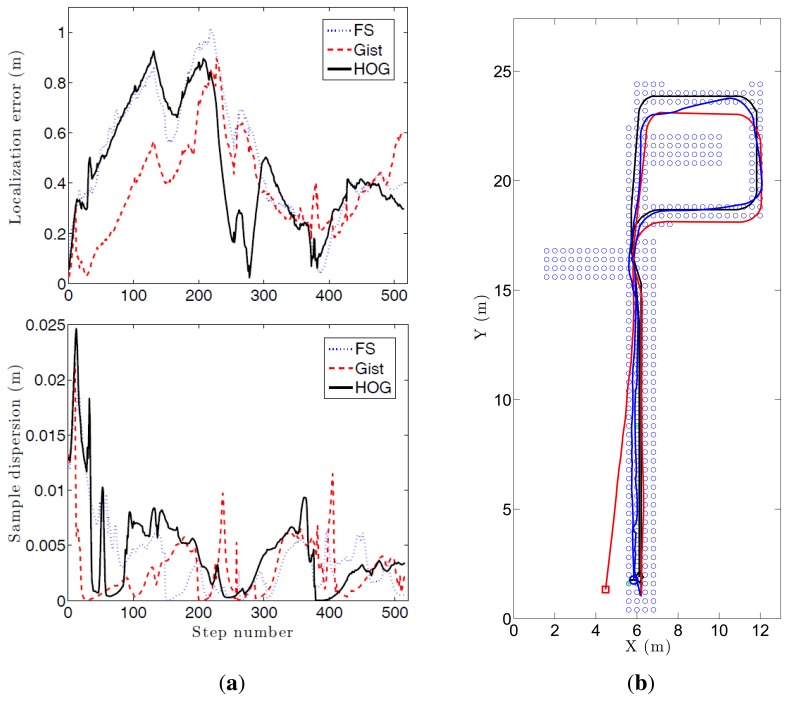
This figure shows the evolution of one of the experiments carried out with the three descriptors: (**a**) evolution of the localization error and the dispersion of the samples and (**b**) bird eye's vies of the process. The blue dots are the positions of the map images, the black curve is the ground truth of the route followed by the robot, the red curve is the trajectory estimated using only the odometry data and the blue curve is the trajectory estimated making use of the probabilistic process.

To carry out this experiment, we make use of part of the *Quorum* database as a map (blue dots in Figure 15b). We have captured a second image set (510 images) while the robot traverses a route within the environment. This second set of images is used to carry out the probabilistic localization. This is a specially challenging problem due to the fact that the second set of images have been captured under different lighting conditions comparing to the first database and this environment is especially prone to visual aliasing.

**Figure 16.**
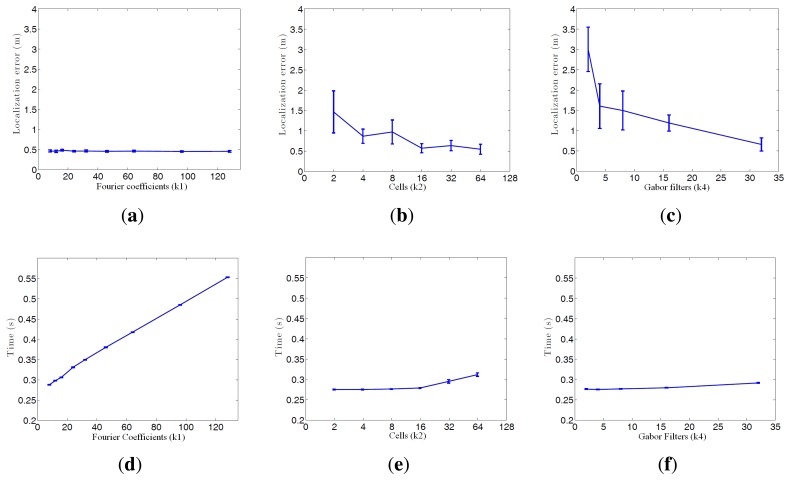
This figure shows the average error during the localization process depending on the descriptor parameters (**a**) Fourier Signature; (**b**) HOG; (**c**) *gist* and the average step time during localization depending on the descriptor parameters (**d**) Fourier Signature, (**e**) HOG; (**f**) *gist*.

Figure 16 shows the average error during the localization process and the step time. Every time a route image arrives and the position of the robot is estimated using the Monte Carlo algorithm is considered a step. If we compute the localization error at each step (comparing the result of the algorithm with the actual position of the robot) we get the curves at Figure 16a–c. These curves show how the behavior of the Fourier Signature is the most stable independently on the value of *k*_1_. HOG presents similar results when the number of cells *k*_2_ is between 16 and 64, and *gist* presents also better results when the number of Gabor masks *k*_4_ is high, but the error in all cases is higher comparing to Fourier Signature.

As far as step time is concerned, *gist* is the quickest algorithm. HOG presents similar results when the number of cells *k*_2_ is lower than 16. Fourier Signature presents a higher computational cost, and it increases as *k*_1_ does.

Figure 15 shows the evolution of three experiments carried out with FS and *k*_1_ = 32, HOG and *k*_2_ = 16, and *gist* and *k*_4_ = 16. (a) shows the localization error and the dispersion of the particles. A sudden increase in this dispersion indicates visual aliasing (the nearest images in the database are in far points). In general, the dispersion is high at the beginning and decreases as new information arrives. The algorithm is able to recover from visual aliasing with the three descriptors. Figure 15b shows a bird's eye view of the process when using the HOG descriptor and *k*_2_ = 16. The blue dots are the positions of the map images, the black curve is the ground truth of the route followed by the robot, the red curve is the trajectory estimated using only the odometry data and the blue curve is the trajectory estimated making use of the probabilistic process. The robot starts at the bottom of the map (coordinates *x* = 6, *y* = 2 m), advances to the upper side of the figure, closes the loop and goes back to the initial position.

### 5.6. Kidnapped Robot Problem in Monte Carlo Localization

Once we have shown how the descriptors behave in a probabilistic localization process under usual working conditions, to conclude with the experiments we test them in the resolution of the kidnapped robot problem. In this problem, a robot which is well localized during a probabilistic process is teleported to a different location without noticing it. This is a very interesting problem as it tests the ability of the localization algorithm to recover from serious localization errors or temporal failures of sensory systems.

To solve this problem with robustness, we have decided to make a slight variation of the Monte Carlo algorithm presented in the precedent section. During the resampling process, we have decided to add a new set of particles at random positions. This set of particles represents a low percentage of the number of particles in the total global set. When the robot is well localized, these random particles are expected not to affect the localization algorithm but, after the kidnapping of the robot, the random particles which are near the new position of the robot are expected to act as a seed that makes the probability distribution to tend to that real position.

In these experiments, 95% of the particles in the new set *χ_t_* come from the resampling of the previous set *χ_t_*_−1_ using the SIR algorithm and the remaining 5% of the particles are sampled from a uniform distribution over the robot's map.

**Figure 17.**
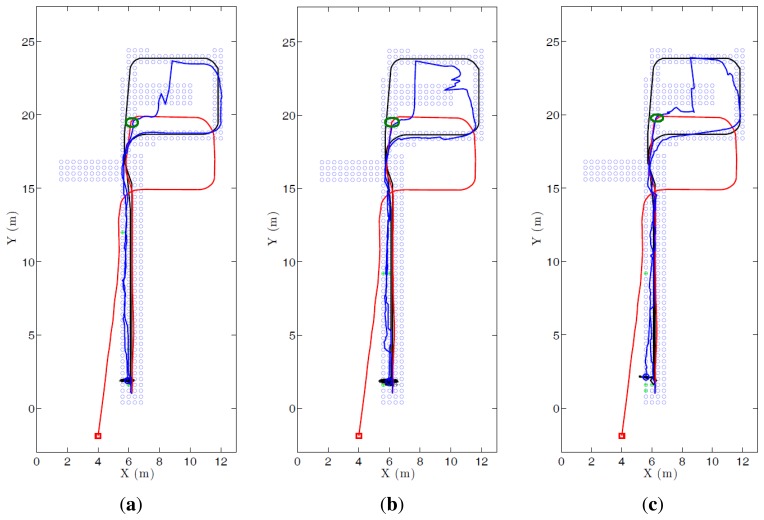
This figure shows the evolution of three kidnapped robot experiments using (**a**) Fourier Signature; (**b**) HOG and (**c**) *gist*. The blue dots are the positions of the map images, the black curve is the ground truth of the route followed by the robot, the red curve is the trajectory estimated using only the odometry data and the blue curve is the trajectory estimated making use of the probabilistic process.

Figure 17 shows the evolution of three kidnapped robot experiments carried out with (a) FS and *k*_1_ = 32; (b) HOG and *k*_2_ = 16; and (c) *gist* and *k*_4_ = 16. In all cases the kidnapping is produced at the same point, during the ascending trajectory (the exact point is marked with a green circle). The descriptor which first recovers from the kidnapping is HOG. However it presents some problems of visual aliasing (see upper right corner in Figure 17b). Fourier Signature and *gist* present a similar behaviour in this experiment. Anyway, the robot is able to recover from the kidnapping in the three cases, closes the loop correctly and localizes with a good accuracy until the final point of the route.

**Figure 18.**
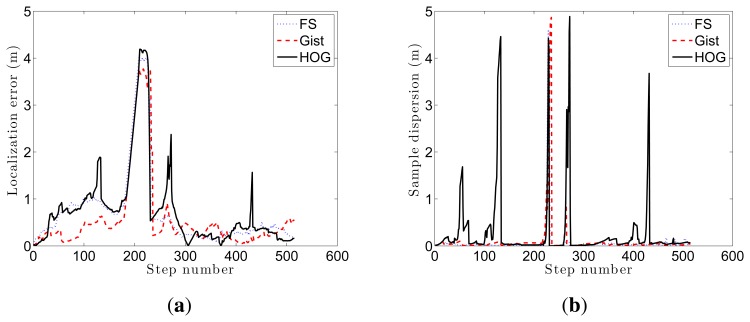
Evolution of the localization error and the sample dispersion in the previous experiments.

Figure 18 shows the evolution of (a) the localization error and (b) the dispersion of the samples during these three experiments. (a) shows a sudden increase in error around step 200, when the robot is kidnapped. It recovers relatively quickly from this error and HOG produces another error around step 280, but it also recovers soon; (b) shows some cases of visual aliasing that produce sudden increases on the dispersion of the particles.

## 6. Discussion

Once we have presented the results, in this section we make a discussion of these results in the three fields we have analyzed: map building, localization and probabilistic localization. We have arrived to some general conclusions about the use of the four description methods. PCA with rotations presents, by far, the higher computational cost during the creation of the map. It makes this process unfeasible to model large environments. Also, comparing to the other three descriptors, PCA is not an incremental method. This means that, if we have created a map with a set of images and we want to add a new image to the map, the mapping process must be started from the scratch. This way, the whole map must be available before starting the localization process. By this reason, this method may be not advisable for certain tasks, such as SLAM (Simultaneous Localization and Mapping). Fourier Signature, HOG and *gist* do not present this disadvantage.

Comparing these three descriptors in a map building task, Fourier Signature needs, in general, more memory and *gist* is the most compact representation. However, *gist* has the heaviest computational cost and Fourier Signature is the quickest process. HOG presents a good compromise between memory and computational cost.

During the localization process, PCA with rotations is the quickest algorithm to estimate position and orientation. HOG is also very quick and Fourier Signature and *gist* present acceptable results when the number of components is low. The precision in the position estimation presents good results when using the algorithms. Fourier Signature, PCA and *gist* present a very good and stable behavior independently on the descriptor size, and HOG presents good results when the number of components is not very high. These results get worse when there is a change in lighting conditions or some parts of the scenes are ocluded. HOG and *gist* present the best results in these cases. However, the Fourier Signature and PCA with rotations present a better behavior when some noise appears in the scenes.

As far as the probabilistic localization process is concerned, the best results have been obtained with the HOG descriptor, as it presents a good compromise between average error and computational cost for an intermediate number of components (between 8 and 16 cells). Fourier Signature presents a good accuracy but the computational cost is higher and the results in accuracy are worse when using *gist*. At last, these three descriptors have been able to solve the robot kidnapping problem with an adequate choice of the parameters.

## 7. Conclusions

In this paper we have studied and compared four approaches to describe panoramic scenes based on their global appearance. The methods we have studied are the Fourier Signature, Principal Components Analysis with Rotations, Histogram of Oriented Gradients and *gist*. We have used these approaches to solve the map building and localization problems using a mobile robot with an omnidirectional vision sensor mounted on it. The main contributions of the paper include the adaptation of the HOG and *gist* descriptors to be used with panoramic images with rotational invariance, the study and optimization of the four methods to create a visual representation and the validation of these maps. This validation has been carried out from three points of view: global localization, robust localization against changes in lighting conditions, occlusions and noise and probabilistic localization. In all cases we have compared the performance of the descriptors and the influence of their main configuration parameters. Due to the increasing use of global appearance methods in mobile robotics we think it was necessary to carry out a deep and exhaustive comparative analysis of the main existing methods. All the experiments have been carried out with two sets of panoramic images captured in different rooms under real working conditions.

The results presented in this paper show the feasibility of global appearance methods in mapping and localization tasks. We are now working on new description methods that improve the localization results, especially under occlusions and changes in lighting conditions, on new mapping methods to include more information about the relationships between positions and on solving the SLAM problem using global appearance.

## Figures and Tables

**Table 1.** Quorum database. Precision (%) in localization when the test images present occlusion or noise.

	**Occlusion Percentage**						**Noise variance**			
	0	5	10	20	40	0	0.01	0.02	0.04	0.08
FS	53	46	40	32	13	53	53	53	53	46
PCA rot. (*)	67	62	54	38	5	67	64	63	63	62
HOG	68	60	54	43	17	68	60	43	33	26
*Gist*	54	42	38	35	20	54	49	45	43	25
